# Synchronous Supraglottic and Esophageal Squamous Cell Carcinomas Treated with a Monoisocentric Hybrid Intensity-Modulated Radiation Technique

**DOI:** 10.3389/fonc.2017.00307

**Published:** 2018-01-08

**Authors:** Christian L. Barney, Pedro Zamora, Ashlee Ewing, Matthew Old, Arnab Chakravarti, Aashish Bhatt

**Affiliations:** ^1^Radiation Oncology, Ohio State University, Columbus, OH, United States; ^2^Head and Neck Surgery, Ohio State University, Columbus, OH, United States

**Keywords:** head and neck cancer, esophageal cancer, squamous cell carcinoma, supraglottic cancer, intensity-modulated radiation, synchronous tumors, monoisocenter

## Abstract

Risk factors for squamous cell carcinomas (SCCs) of the head and neck (HN) and esophagus are similar. As such, synchronous primary tumors in these areas are not entirely uncommon. Definitive chemoradiation (CRT) is standard care for locally advanced HNSCC and is a preferred option for inoperable esophageal SCC. Simultaneous treatment of both primaries with CRT can present technical challenges. We report a case of synchronous supraglottic and esophageal SCC primary tumors, highlighting treatment with a monoisocentric hybrid radiation technique and normal tissue toxicity considerations.

## Introduction

Despite the continued decline in incidence of esophageal squamous cell carcinoma (ESCC) in the United States, the rate of synchronous primary head and neck squamous cell carcinoma (HNSCC) and ESCC remains relatively unchanged over the past two decades (1, 2). The presence of a second primary malignancy can bring with it unique therapeutic challenges and has been associated with inferior clinical outcomes (3, 4). Curative chemoradiation (CRT) is an effective treatment for isolated locally advanced HNSCC and is a next-best alternative therapy in non-operable ESCC (5, 6). Unfortunately, simultaneous CRT treatment of these tumors necessitates the delivery of high-dose radiation to expansive clinical target volumes (CTVs), causing concern for high rates of normal tissue toxicity. In such cases, an eloquent, unified radiotherapy (RT) plan with a single isocenter can help to optimize dose distributions. This report highlights a unique case of synchronous supraglottic and ESCCs treated simultaneously with CRT using a monoisocentric hybrid 3D-conformal/intensity-modulated radiation (IMRT) technique.

## Background

**Figure 1:**
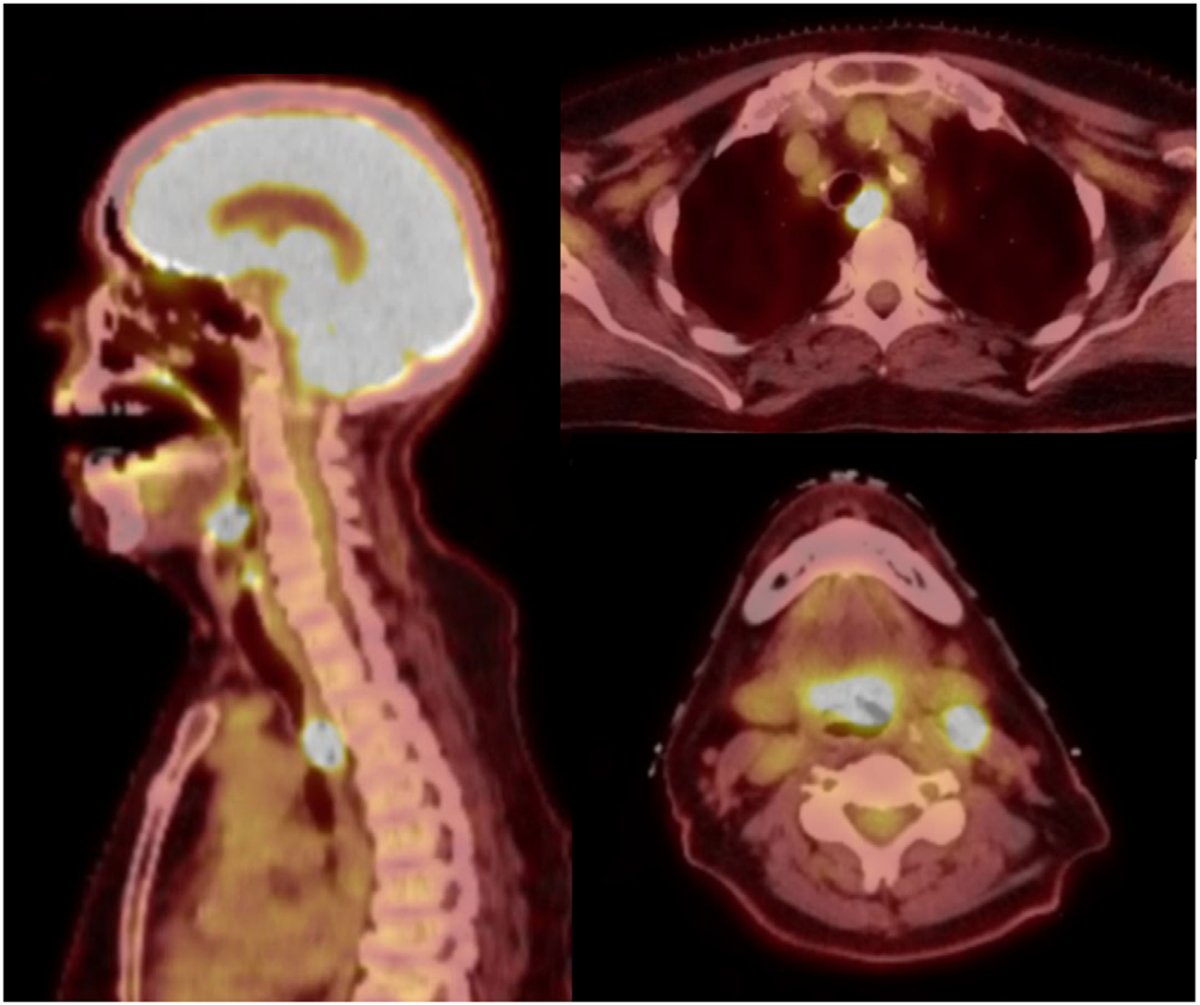
Midline sagittal and axial views on positron emission tomography-computerized tomography. There are foci of hyperavidity in the supraglottic larynx, level II cervical lymph nodes, and the upper thoracic esophagus.

A 76-year-old female presented with persistent throat pain and mild dysphagia. A midline lesion of the laryngeal epiglottis with vallecular extension was noted on laryngoscopy exam, the bilateral true vocal cords were normal in appearance and mobility. Biopsy of the mass showed HPV-negative squamous cell carcinoma (SCC). Subsequent computerized tomography neck and chest showed bilateral level II cervical lymphadenopathy. Staging positron emission tomography scan demonstrated activity in the supraglottic mass, bilateral level II cervical lymph nodes (LNs), and unexpectedly within the upper esophagus (Figure 1). Endoscopy with ultrasound noted a mucosa-confined esophageal mass, extending from 20 to 22 cm from the incisors. A pathologically enlarged level 2L LN was also noted, but was not amenable to sampling. Biopsy of the esophageal mass showed invasive SCC and the patient was ultimately diagnosed with cT3N2cM0 (stage IVA) and uT1bN1M0 (stage IIB) supraglottic and esophageal cancers, respectively. Interdisciplinary tumor board recommendation was to proceed with definitive CRT for both malignancies given the patient’s age and medical comorbidities.

### RT Planning and Chemotherapy

**Figure 2:**
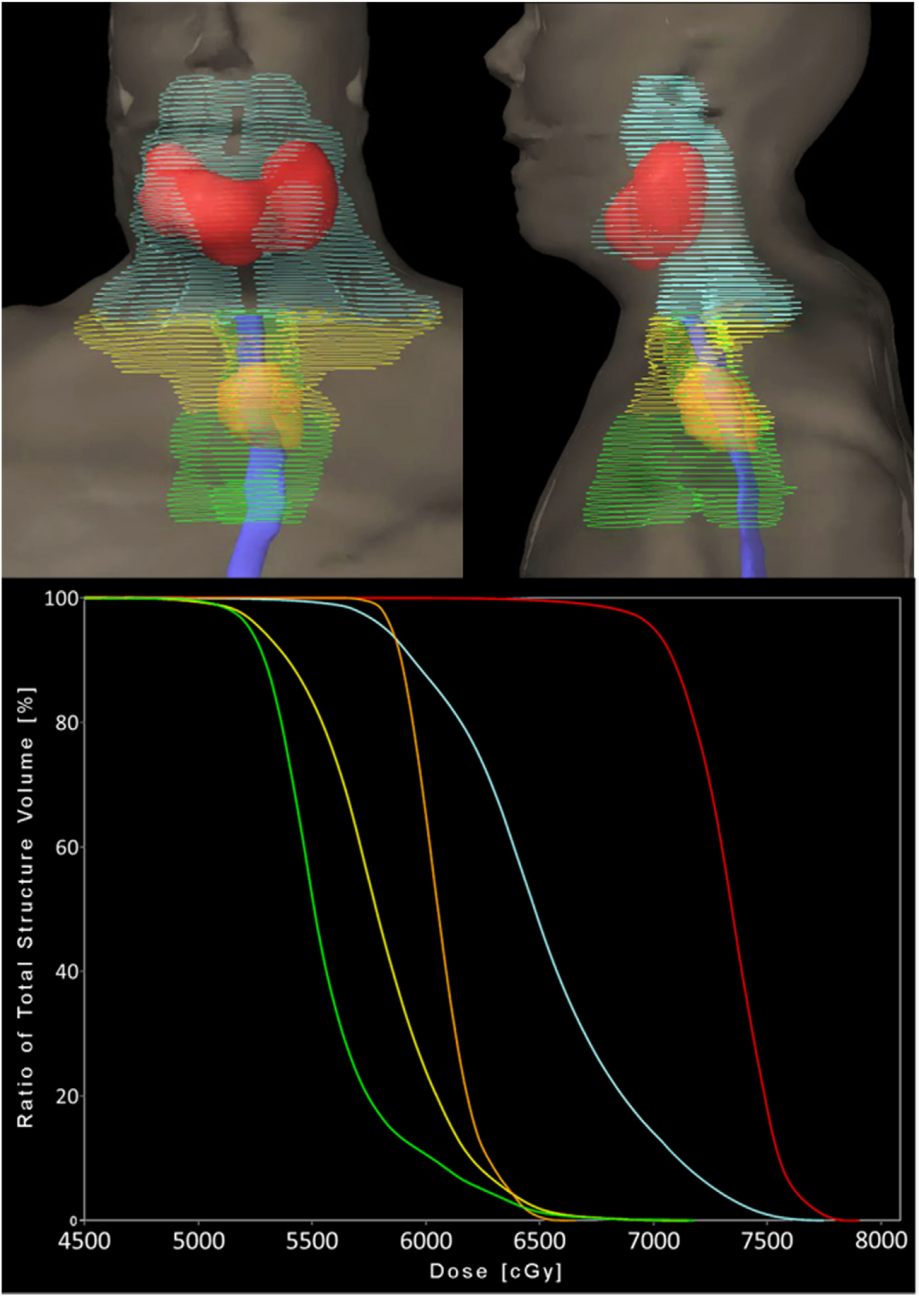
Planning target volumes and respective dose–volume histogram. Coronal and sagittal 3D volume renderings (top) by color: orange = esophageal primary, green = elective esophageal lymph nodes (LNs), yellow = bilateral low-risk elective cervical LNs, cyan = bilateral intermediate-risk elective cervical LNs, and red = supraglottic primary and gross nodal disease. Solid blue = esophagus contour. Each color is represented as a structure on the dose–volume histogram (bottom).

**Figure 3:**
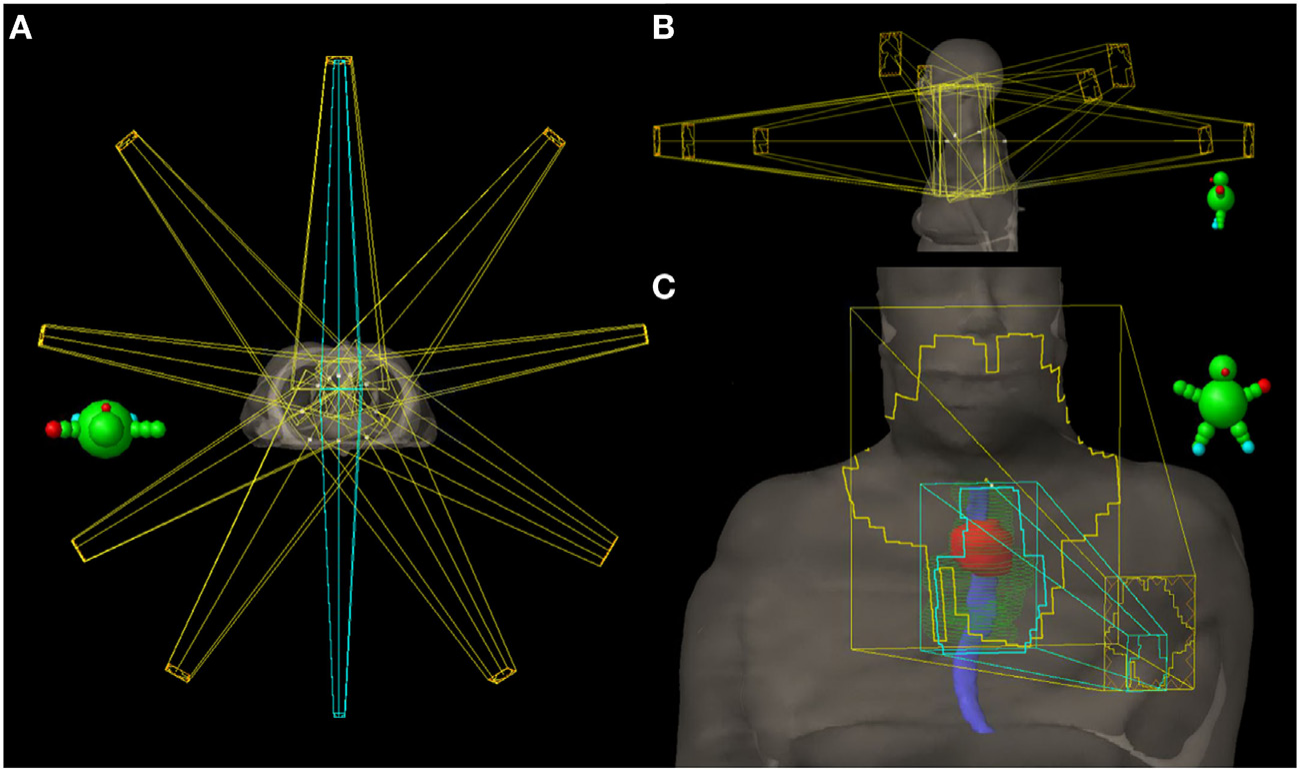
Radiation treatment plan. **(A)** Axial view of all 11 fields with the 3D-conformal AP/PA fields highlighted in cyan. **(B)** Sagittal views of the nine intensity-modulated radiation (IMRT) fields—five coplanar beams and four additional non-coplanar beams which have been angled to reduce entrance and exit dose in normal tissue. **(C)** AP view showing the AP 3D-conformal (cyan) and IMRT (yellow) fields and their body surface projections. The 3D-conformal field is contained within the AP IMRT field’s fluence and is specifically targeting the esophageal primary (red contour) and elective esophageal nodes (green contour). Solid blue = esophagus contour.

**Figure 4:**
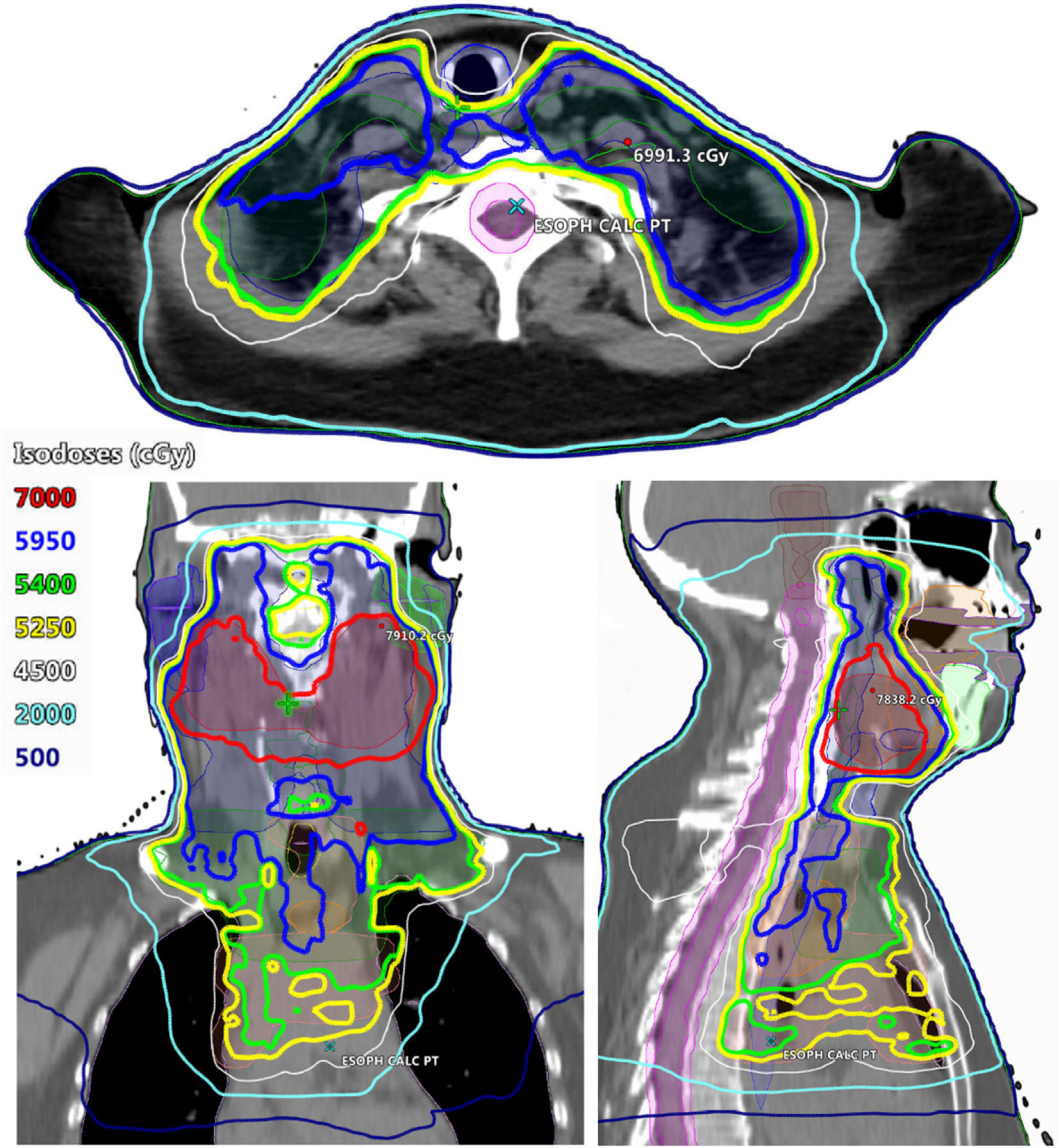
Radiation isodose distributions. (Top) Axial view at the level of the subglottic larynx. (Bottom left) Coronal view. (Bottom right) Midline sagittal view.

Radiotherapy treatment planning goals were achieved for target and normal tissue structures (Figure 2; Table 1) using a monoisocentric, hybrid 3D-conformal/IMRT technique (Figure 3), including nine static IMRT beams and opposed low 3D-conformal AP/PA beams. The AP/PA beams were used as a base plan, allowing those beams to be fully taken into account by the optimizer during subsequent IMRT beam planning to avoid field junction heterogeneity. Doses were prescribed to the following planning target volumes: elective esophageal LNs (52.5 Gy), low-risk elective neck (54.0 Gy), esophageal primary and intermediate-risk elective neck (59.5 Gy), and supraglottic primary/high-risk gross nodal disease (70 Gy) (Figure 3). RT was delivered over 7 weeks in five identical daily fractions per week using a simultaneous integrated boost with weekly concurrent carboplatin (AUC = 2) and paclitaxel (50 mg/m^2^). Isodose distributions are shown in Figure 4.

**Table 1:** Normal structure dosimetry.

Structure	Mean (Gy)	Max (Gy)	Other
Esophagus	36.3	66.6	
Lung	15.9	63.6	V5 = 67.9%
			V20 = 28.8%
Brachial plexus	56.4	69.6	
Constrictors	65.1	78.0	
Parotids			
Left	20.8		
Right	19.9		

### Response to Treatment and Toxicity

Acute and late toxicities were assessed using radiation therapy oncology group (RTOG) toxicity criteria. The patient developed acute grade 3 dermatitis, mucositis, and dysphagia [percutaneous endoscopic gastrostomy (PEG) tube placed in week 3 of CRT]; however, no RT breaks were required. At a follow-up time of 34 months, the patient is without clinical, endoscopic, or radiographic evidence of malignancy. She has developed several long-term treatment-related toxicities including grade 2 xerostomia and neck soft tissue fibrosis in addition to grade 3 esophageal stricturing with near complete stenosis. She has undergone five esophageal dilations since treatment conclusion—initially requiring retrograde and anterograde dilation from her PEG and mouth sites, respectively. She is limited to oral soft foods and infrequently supplements her nutrition *via* PEG tube when needed.

## Discussion

There are various treatment strategies in patients with synchronous ESCC and HNSCC, including different combinations of surgery, chemotherapy, and radiation. A recent retrospective study of 91 patients treated for synchronous head and neck (HN) and esophageal SCC showed that both upfront simultaneous resection or definitive CRT are reasonable treatment options, with 3-year survival rates of 67 vs 49%, respectively. However, definitive CRT was associated with a higher rate of grade 3–5 adverse events and 47% required salvage surgery for residual or recurrent HN disease (7). In non-surgical candidates, definitive CRT has proven to be a viable treatment option for isolated ESCC, with 2-year local control (LC) rates of 41–57% (5, 8). It is also an effective organ preservation therapy in isolated locally advanced laryngeal SCC (6). However, long-term toxicity rates remain high for CRT in both of these primary sites. For example, long-term results of the RTOG 91-11 trial showed grade ≥3 late esophageal and laryngeal toxicity rates of 17 and 15%, respectively, in patients treated with CRT for locally advanced laryngeal SCC. In esophageal CRT, Cooper et al. reported grade ≥3 late toxicity rates of 29%, most of which (22%) directly involved the esophagus (5). Unfortunately, these toxicities can significantly affect quality of life as illustrated by this case report. A theoretic increase in these toxicities should be expected when treating synchronous tumors given the larger radiation fields required to adequately cover both sites; as such, avenues to maximize the therapeutic ratio should be considered in such cases.

The extent of high-dose RT fields should be of primary consideration. Because of the rich lymphatic network of the esophagus, a large longitudinal field expansion around the esophageal primary is required (3–5 cm superiorly and inferiorly) to encompass microscopic disease (9), as such most CRT protocols are now favoring an involved nodal approach in an effort to reduce field size elsewhere. In our patient, we were more aggressive with elective nodal volumes, treating not only level 2L (the area of clinical involvement) but also thoracic levels 2–8 (level 8 to 5 cm beyond the inferior extent of the primary CTV); the bilateral supraclavicular regions were covered in the HN nodal CTVs. In retrospect, an involved nodal approach for the esophagus may have reduced toxicity, although we cannot predict how this would have impacted locoregional control.

Selection of RT dose and fractionation schedule is also crucial in striking a balance between tumor control and toxicity. Delivery of 70 Gy to gross tumor over 7 weeks in 2-Gy daily fractions with cisplatin chemotherapy is a standard curative regimen in locally advanced HNSCCs. Given the larger field sizes, the use of altered fractionation should be approached with caution when treating synchronous tumors, especially if concurrent chemotherapy is given or if the patient is elderly (as in our case) or of poor performance status (KPS < 80) (10, 11). In such cases, the increased acute toxicity rates experienced with altered fractionation may outweigh potential LC benefits.

For ESCC, the results of early trials led to the adoption of 50.4 Gy in 28 fractions as the standard for definitive CRT (5, 12). Specifically, the INT 0123 trial was a CRT dose escalation study (64.8 vs 50.4 Gy) that showed no improvement in LC (50 vs 55%) and an increased treatment-related mortality (10 vs 2%, 11 vs 2 patients) in the high-dose arm, although 7 of 11 events occurred before reaching doses of 50.4 Gy (12). Some investigators attribute these results to antiquated radiation techniques and the use of lower dose 5-FU in the 64.8 Gy arm (13). These uncertainties and continued poor LC rates have prompted further exploration of dose escalation. Our decision to dose escalate was driven by retrospective studies that suggest higher doses (51–65 Gy) likely improve LC and correlate positively with pathologic complete response (pCR) rates in ESCC (14–17); however, one study did report increased esophageal stricture rates with doses >50.4 Gy (32.1 vs 18.2%) (16). In our patient, we followed the chemotherapy regimen used in the CROSS trial given the high ESCC pCR rates in that study (48%) and reports of carbo/paclitaxel being an effective alternative in HNSCC when patients are cisplatin ineligible (18–20).

The selection of RT technique can be just as important as that of dose/fractionation and chemotherapeutic regimen. Dose-painting IMRT in HNSCC has proven to improve quality of life and reduce long-term toxicity rates (21). The benefits of IMRT in ESCC are less clear—comparisons to 3D-conformal plans show superior homogeneity and dose–volume parameters in the lungs and heart, although the clinical significance of these improvements is unknown (22). In planning our patient, IMRT alone was optimal for target–dose conformity and homogeneity; however, the elevated low-dose bath to the lungs would have placed the patient at high risk for severe pneumonitis. In this scenario, the use of AP/PA conformal beams has been shown to reduce the low-dose lung bath by forcing dose centrally within the thorax, as was the case in our patient (23). We applied this technique in our patient and accordingly were able to reduce the lung dose to within acceptable constraints. The downsides of a hybrid technique, however, are competing cardiac toxicity and mildly increased dose heterogeneity. Dose heterogeneity can also be introduced at field junctions. A monoisocentric technique is thus desirable in an effort to improve dose distributions (24) and avoid junctional “hot” and “cold” spots, as was highlighted in this case report.

## Concluding Remarks

This unique case highlights the utility of monoisocentric and hybrid IMRT techniques for treating synchronous supraglottic and esophageal SCCs. In this setting, field size and dose/fractionation selections are integral in striking a balance between toxicity and tumor control. Despite these considerations, severe long-term treatment-related toxicities, including esophageal stricture, are not entirely unavoidable as illustrated in this report.

## Informed Consent

The patient included in this case report provided her verbal and written/signed consent to submit a report of her de-identified cancer treatment history and related toxicities for publication in a publically available journal.

## Ethics Statement

This case report was written in accordance with the recommendations of the Office of Responsible Research Practices at our institution and is covered under our institutional review board (IRB) committee approved protocol 2015C012. Verbal and written/signed informed consent was obtained from the patient discussed in this case.

## Author Contributions

Conceptualization and writing—review and editing: CB, PZ, AC, AB, AE, and MO; investigation: CB, PZ, and AE; writing—original draft: CB and PZ; visualization: CB and AE; project administration: CB, MO, AC, and AB; supervision: MO, AC, and AB.

## Conflict of Interest Statement

The authors declare that the research was conducted in the absence of any commercial or financial relationships that could be construed as a potential conflict of interest.

## References

[1] Jain KS Sikora AG Baxi SS Morris LGT Synchronous cancers in patients with head and neck cancer. Cancer (2013) 119:1832–7. 10.1002/cncr.27988 23423883

[2] McGarey PO O’Rourke AK Owen SR Shonka DC Reibel JF Levine PA Rigid esophagoscopy for head and neck cancer staging and the incidence of synchronous esophageal malignant neoplasms. JAMA Otolaryngol Head Neck Surg (2016) 142:40–5. 10.1001/jamaoto.2015.2815 26633039

[3] Lee JS Ahn JY Choi KD Song HJ Kim YH Lee GH Synchronous second primary cancers in patients with squamous esophageal cancer: clinical features and survival outcome. Korean J Intern Med (2016) 31:253–9. 10.3904/kjim.2014.182 26864297 PMC4773710

[4] Li Q-W Zhu Y-J Zhang W-W Yang H Liang Y Hu Y-H Chemoradiotherapy for synchronous multiple primary cancers with esophageal squamous cell carcinoma: a case-control study. J Cancer (2017) 8:563–9. 10.7150/jca.17408 28367236 PMC5370500

[5] Cooper JS Guo MD Herskovic A Macdonald JS Martenson JA Jr Al-Sarraf M Chemoradiotherapy of locally advanced esophageal cancer: long-term follow-up of a prospective randomized trial (RTOG 85-01). JAMA (1999) 281:1623–7. 10.1001/jama.281.17.1623 10235156

[6] Forastiere AA Zhang Q Weber RS Maor MH Goepfert H Pajak TF Long-term results of RTOG 91-11: a comparison of three nonsurgical treatment strategies to preserve the larynx in patients with locally advanced larynx cancer. J Clin Oncol (2013) 31:845–52. 10.1200/JCO.2012.43.6097 23182993 PMC3577950

[7] Morita M Egashira A Nakaji Y Kagawa M Sugiyama M Yoshida D Treatment of squamous cell carcinoma of the esophagus synchronously associated with head and neck cancer. In Vivo (2017) 31:909–16. 10.21873/invivo.11146 28882958 PMC5656865

[8] Bedenne L Michel P Bouché O Milan C Mariette C Conroy T Chemoradiation followed by surgery compared with chemoradiation alone in squamous cancer of the esophagus: FFCD 9102. J Clin Oncol (2007) 25:1160–8. 10.1200/JCO.2005.04.7118 17401004

[9] Gao X Qiao X Wu F Cao L Meng X Dong Z Pathological analysis of clinical target volume margin for radiotherapy in patients with esophageal and gastroesophageal junction carcinoma. Int J Radiat Oncol Biol Phys (2007) 67:389–96. 10.1016/j.ijrobp.2006.09.015 17236963

[10] Pignon J-P le Maître A Bourhis J Meta-analyses of chemotherapy in head and neck cancer (MACH-NC): an update. Int J Radiat Oncol Biol Phys (2007) 69:S112–4. 10.1016/j.ijrobp.2007.04.088 17848275

[11] Bonner JA Harari PM Giralt J Cohen RB Jones CU Sur RK Radiotherapy plus cetuximab for locoregionally advanced head and neck cancer: 5-year survival data from a phase 3 randomised trial, and relation between cetuximab-induced rash and survival. Lancet Oncol (2010) 11:21–8. 10.1016/S1470-2045(09)70311-0 19897418

[12] Minsky BD Pajak TF Ginsberg RJ Pisansky TM Martenson J Komaki R INT 0123 (radiation therapy oncology group 94-05) phase III trial of combined-modality therapy for esophageal cancer: high-dose versus standard-dose radiation therapy. J Clin Oncol (2002) 20:1167–74. 10.1200/JCO.20.5.1167 11870157

[13] Willett CG Radiation dose escalation in combined-modality therapy for esophageal cancer. J Clin Oncol (2002) 20:1151–3. 10.1200/JCO.2002.20.5.1151 11870151

[14] Wang S Liao Z Chen Y Chang JY Jeter M Guerrero T Esophageal cancer located at the neck and upper thorax treated with concurrent chemoradiation: a single-institution experience. J Thorac Oncol (2006) 1:252–9. 10.1016/S1556-0864(15)31576-8 17409865

[15] Zhang Z Liao Z Jin J Ajani J Chang JY Jeter M Dose-response relationship in locoregional control for patients with stage II-III esophageal cancer treated with concurrent chemotherapy and radiotherapy. Int J Radiat Oncol Biol Phys (2005) 61:656–64. 10.1016/j.ijrobp.2004.06.022 15708243

[16] He L Allen PK Potter A Wang J Chang JY Gomez DR Re-evaluating the optimal radiation dose for definitive chemoradiotherapy for esophageal squamous cell carcinoma. J Thorac Oncol (2014) 9:1398–405. 10.1097/JTO.0000000000000267 25122435

[17] Geh JI Bond SJ Bentzen SM Glynne-Jones R. Systematic overview of preoperative (neoadjuvant) chemoradiotherapy trials in oesophageal cancer: evidence of a radiation and chemotherapy dose response. Radiother Oncol (2006) 78:236–44. 10.1016/j.radonc.2006.01.009 16545878

[18] van Hagen P Hulshof MCCM van Lanschot JJB Steyerberg EW van Berge Henegouwen MI Wijnhoven BPL Preoperative chemoradiotherapy for esophageal or junctional cancer. N Engl J Med (2012) 366:2074–84. 10.1056/NEJMoa1112088 22646630

[19] Behera M Owonikoko TK Kim S Chen Z Higgins K Ramalingam SS Concurrent therapy with taxane versus non-taxane containing regimens in locally advanced squamous cell carcinomas of the head and neck (SCCHN): a systematic review. Oral Oncol (2014) 50:888–94. 10.1016/j.oraloncology.2014.06.014 25060589

[20] Suntharalingam M Haas ML Conley BA Egorin MJ Levy RNS Sivasailam S The use of carboplatin and paclitaxel with daily radiotherapy in patients with locally advanced squamous cell carcinomas of the head and neck. Int J Radiat Oncol Biol Phys (2000) 47:49–56. 10.1016/S0360-3016(00)00408-9 10758304

[21] O’Sullivan B Rumble RB Warde P Members of the IMRT Indications Expert Panel. Intensity-modulated radiotherapy in the treatment of head and neck cancer. Clin Oncol (2012) 24:474–87. 10.1016/j.clon.2012.05.006 22770590

[22] Kole TP Aghayere O Kwah J Yorke ED Goodman KA. Comparison of heart and coronary artery doses associated with intensity-modulated radiotherapy versus three-dimensional conformal radiotherapy for distal esophageal cancer. Int J Radiat Oncol Biol Phys (2012) 83:1580–6. 10.1016/j.ijrobp.2011.10.053 22284687

[23] Mayo CS Urie MM Fitzgerald TJ Ding L Lo YC Bogdanov M. Hybrid IMRT for treatment of cancers of the lung and esophagus. Int J Radiat Oncol Biol Phys (2008) 71:1408–18. 10.1016/j.ijrobp.2007.12.008 18262730

[24] Ahmad M Nath R. Three-dimensional radiotherapy of head and neck and esophageal carcinomas: a monoisocentric treatment technique to achieve improved dose distributions. Int J Cancer (2001) 96:55–65. 10.1002/1097-0215(20010220)96:1<55:AID-IJC6>3.0.CO;2-# 11241330

